# Transcriptomic profiling of sporadic Alzheimer’s disease patients

**DOI:** 10.1186/s13041-022-00963-2

**Published:** 2022-10-12

**Authors:** Andrew B. Caldwell, Balaji G. Anantharaman, Srinivasan Ramachandran, Phuong Nguyen, Qing Liu, Ivy Trinh, Douglas R. Galasko, Paula A. Desplats, Steven L. Wagner, Shankar Subramaniam

**Affiliations:** 1 Department of Bioengineering, University of California, San Diego, La Jolla, CA USA; 2 Department of Neurosciences, University of California, San Diego, La Jolla, CA USA; 3 Present Address: Department of Obstetrics, Gynecology, and Reproductive Sciences, University of California, San Diego, La Jolla, CA USA; 4 Present Address: Department of Microbiology and Immunology, Tulane University School of Medicine, New Orleans, LA USA; 5 Department of Pathology, University of California, San Diego, La Jolla, CA USA; 6 VA San Diego Healthcare System, La Jolla, CA USA; 7 Department of Cellular and Molecular Medicine, University of California, San Diego, La Jolla, CA USA; 8 Department of Nanoengineering, University of California, San Diego, La Jolla, CA USA; 9 Department of Computer Science and Engineering, University of California, San Diego, La Jolla, CA USA

## Abstract

Alzheimer’s disease (AD) manifested before age 65 is commonly referred to as early-onset AD (EOAD) (Reitz et al. Neurol Genet. 2020;6:e512). While the majority (> 90%) of EOAD cases are not caused by autosomal-dominant mutations in *PSEN1*, *PSEN2*, and *APP*, they do have a higher heritability (92–100%) than sporadic late-onset AD (LOAD, 70%) (Wingo et al. Arch Neurol. 2012;69:59–64, Fulton-Howard et al. Neurobiol Aging. 2021;99:101.e1–101.e9). Although the endpoint clinicopathological changes, i.e., Aβ plaques, tau tangles, and cognitive decline, are common across EOAD and LOAD, the disease progression is highly heterogeneous (Neff et al. Sci Adv Am Assoc Adv Sci. 2021;7:eabb5398). This heterogeneity, leading to temporally distinct age at onset (AAO) and stages of cognitive decline, may be caused by myriad combinations of distinct disease-associated molecular mechanisms. We and others have used transcriptome profiling in AD patient-derived neuron models of autosomal-dominant EOAD and sporadic LOAD to identify disease endotypes (Caldwell et al. Sci Adv Am Assoc Adv Sci. 2020;6:eaba5933, Mertens et al. Cell Stem Cell. 2021;28:1533–1548.e6, Caldwell et al. Alzheimers Demen. 2022). Further, analyses of large postmortem brain cohorts demonstrate that only one-third of AD patients show hallmark disease endotypes like increased inflammation and decreased synaptic signaling (Neff et al. Sci Adv Am Assoc Adv Sci. 2021;7:eabb5398). Areas of the brain less affected by AD pathology at early disease stages**—**such as the primary visual cortex**—**exhibit similar transcriptomic dysregulation as those regions traditionally affected and, therefore, may offer a view into the molecular mechanisms of AD without the associated inflammatory changes and gliosis induced by pathology (Haroutunian et al. Neurobiol Aging. 2009;30:561–73). To this end, we analyzed AD patient samples from the primary visual cortex (19 EOAD, 20 LOAD) using transcriptomic signatures to identify patient clusters and disease endotypes. Interestingly, although the clusters showed distinct combinations and severity of endotypes, each patient cluster contained both EOAD and LOAD cases, suggesting that AAO may not directly correlate with the identity and severity of AD endotypes.

**Supplementary Information:**

The online version contains supplementary material available at 10.1186/s13041-022-00963-2.

## Results and discussion

**Fig. 1:**
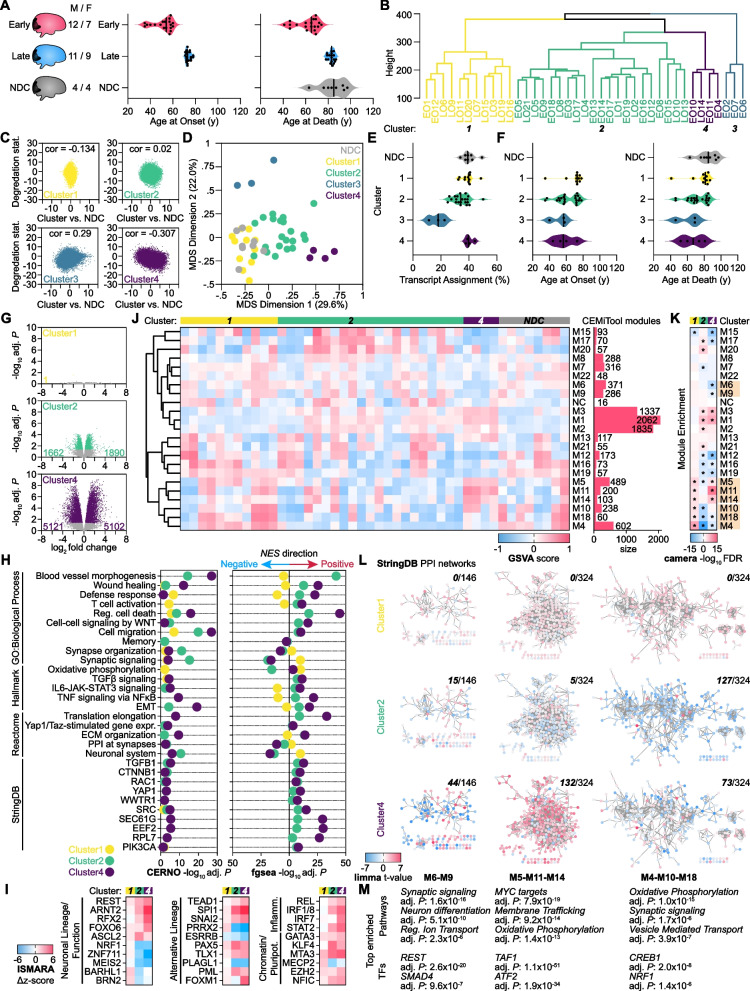
**A** Sex, age at onset (AAO), and age at death (AAD) of early-onset sporadic AD (onset age < 60 years), late-onset sporadic AD (onset age > 70 years), and nondemented control (NDC) patient Occipital Lobe samples. **B** Cluster dendrogram of all AD samples based on the expression of genes (8934) with 10 cpm across all samples. **C** DEqual plot of correlation between differential expression (relative to NDC) and reference patient brain RNA degradation in the 4 clusters. **D** Multi-Dimensional Scaling (MDS) plot of all patient samples for the top two dimensions. **E** Transcriptome assignment % (kallisto) across the sample groups. **F** AAO (left) and AAD (right) across the sample groups. **G** RNA-seq Volcano plots for the three AD clusters. Left, downregulated DEGs; right, upregulated DEGs. **H** Ranked enrichment analysis of gene expression signatures for the three AD clusters using the GOBP, Hallmark, Reactome, and StringDB databases by the *tmod* CERNO (left) and *fgsea* enrichment test (right); plotted data indicates adj. *P* < 0.05. **I** TFs with predicted significant activity change by ISMARA motif analysis curated into canonical ontological categories; [z-score] > 2 in at least one cluster shown. **J** GSVA heatmap, dendrogram, and gene size of the 22 co-expression modules identified by CEMiTool across the sample groups. **K** Camera enrichment analysis of the 22 co-expression modules in the three clusters relative to NDC; * = adj. *P* < 0.05. **L** StringDB PPI interaction networks for ontologically- and expression-related comodules across the three clusters; genes color-coded by limma t-value; upper right subpanel indicates number of DEGs within the comodule for each AD cluster. **M** Top enriched pathways and TFs for each comodule (hypergeometric test)

We sought to identify and characterize molecular phenotypes, or endotypes, associated with Early-onset sporadic (EOS; i.e., cases not caused by autosomal-dominant mutations) Alzheimer’s disease in contrast with Late-onset sporadic (LOS) Alzheimer’s disease using RNA-seq. We selected 40 AD samples from the UC San Diego Shiley-Marcos Alzheimer’s Disease Research Center (ADRC) brain bank stratified into two groups based on the age at onset (AAO) (< 60 years, EOS, 19 samples; > 70 years, LOS, 20 samples after quality filtering) (Fig. 1A). Eight aged, nondemented controls (NDC) were included for comparison. Tissue from the primary visual cortex (Brodmann Area 17, Bm-17) was used for two key reasons: firstly, atypical clinical symptoms, such as visual impairment, are more frequent in EOAD than LOAD; secondly, it mirrors the transcriptomic signature of traditionally affected brain regions without inflammation and gliosis associated with amyloid beta deposition [1, 2]. This provides a unique opportunity to understand the AD transcriptomic signature without confounding factors. Following RNA-seq, hierarchical clustering using scaled gene expression signatures and Euclidean distance to compute pairwise similarity scores failed to dichotomize the samples as early-onset sporadic (EOS) and late-onset sporadic (LOS). Instead, we observed four clusters, only one of which did not have a mixed membership of EOS and LOS cases (Fig. 1B). As the samples did not cluster based on their AAO, we decided to proceed with the four clusters based on transcriptional profiles. Given that frozen postmortem brain samples are prone to RNA degradation, we tested the correlation between differential expression (DE) in each cluster with a previously generated reference brain degradation dataset by generating DEqual plots from the quality surrogate variable analysis (qSVA) framework (Fig. 1C) [3]. By this approach, clusters 1, 2, and 4 showed either no or negative correlation between the degradation and AD-induced DE *t* statistic, whereas cluster 3 showed a positive correlation. Additionally, cluster 3 demonstrated divergence from all other samples in MDS space (Fig. 1D) and had the lowest RNA-seq transcript assignment percentage (Fig. 1E). Therefore, we excluded cluster 3 from further analysis. Interestingly, clusters 1, 2, and 4 did not show a statistically significant difference in AAO or age at death (AAD), although cluster 4 had the earliest AAO and AAD (Fig. 1F). The number of differentially expressed gene transcripts (DEGs) relative to NDC increased from cluster to cluster as the AAO and AAD decreased, with cluster 4 displaying the largest number of DEGs (Fig. 1G). Functional enrichment analysis using the *fgsea* and CERNO with the GO: Biological Process, Reactome, and Hallmark databases (Fig. 1H) revealed gene sets related to dedifferentiation and non-ectoderm lineage definition, inflammation, synaptic function, and oxidative phosphorylation. Enrichment using the StringDB v10 database demonstrated activation of genes that are protein–protein interaction (PPI) partners with TGFB signaling (TGFB1, CTNNB1), transcription factors (TFs) which activate EMT/dedifferentiation (YAP1, WWTR1/TAZ), as well as proteins previously implicated in AD (SRC, SEC61G, EEF2, RPL7) [4–6]. Next, we used ISMARA motif activity analysis to find TFs with differential activity across AD clusters. This revealed activation of TFs controlling early-stage neural lineage commitment or repression of neuron specification and function (REST), repression of neuronal mitochondrial energy production (NRF1) and other neural factors (MEIS2, ZNF711) (Fig. 1I). Further, TFs involved in non-ectoderm and precursor lineage (TEAD1, SPI1/Pu.1, SNAI2), inflammation (REL, IRF1/8), chromatin modification (EZH2, MTA3, MECP2), and pluripotency (KLF4, GATA3) were also enriched, particularly in cluster 4. Next, we sought to identify co-expressed gene modules differentially regulated across the AD clusters. We performed module detection using the *CEMiTool* R package for all genes commonly expressed in AD and NDC samples with > 10 cpm normalized expression [7]. 22 co-expression modules and 1 non-correlated module were identified from the 9120 genes, ranging from 48 to 2062 genes in size. Enrichment scores for each co-expressed module were calculated for each AD and NDC sample using Gene Set Variation Analysis (GSVA) in the *GSVA* R package (Fig. 1J) [8]. The ontological identity of each module was characterized by hypergeometric enrichment of module genes with GO: BP, Reactome, and Hallmark databases (Additional file 1) and their statistical significance by enrichment with camera [6] method. This revealed activation of modules functionally associated with non-ectoderm dedifferentiation and early neurogenesis (Additional file 2: M1; Additional file 1: Fig. S1) and chromatin modification (Additional file 2: M2, M8), as well as repression of modules associated with neuron lineage and function (Additional file 2: M4, M6, M9), and oxidative phosphorylation (Additional file 2: M4, M10, M18), particularly in clusters 2 and 4 (Fig. 1K). Using the similarity dendrogram of module GSVA scores across all samples, we merged individual modules into comodules based on GSVA score similarity and common ontological categories. Three comodules in particular**—** M6–M9, M5–M11–M14, and M4–M10–M18**—**had closely related cellular functions and stratified the three AD clusters (Fig. 1L, M; Additional file 1: Fig. S2). Comodule M6–M9 was significantly enriched for synaptic signaling and neuron differentiation with the neural repressor REST and non-ectoderm lineage factor SMAD4 as key regulators (Additional file 1: Fig. S2). M6-M9 genes were substantially downregulated in cluster 4, modestly downregulated in cluster 2, and mixed regulation in cluster 1. In contrast, comodule M5–M11–M14—which contains genes involved in cell cycle and proliferation (MYC targets), membrane trafficking, and oxidative phosphorylation—was upregulated in clusters 1 and 4 but downregulated in cluster 2 (Additional file 1: Fig. S2). Interestingly, comodule M4–M10–M18, whose top transcriptional regulators are NRF1 and CREB1, is also enriched for oxidative phosphorylation and synaptic signaling as observed in the two other comodules (Additional file 1: Fig. S2). However, M4–M10–M18 genes were most downregulated in cluster 2, suggesting that while loss of synaptic function and oxidative phosphorylation are common between clusters 2 and 4, distinct pools of genes are differentially regulated in the two clusters. Further, their expression loss is mediated by unique regulators (e.g., gain of repression by REST versus loss of activation by NRF1).

At the outset of this study, we aimed to characterize the relationship between the severity of AD endotype dysregulation and Age at Onset (AAO). However, we quickly determined that the patient transcriptional profiles in our AD cohort did not bifurcate into early-onset (EOS) and late-onset (LOS) cases. Following filtering, three clusters were identified with differing ratios of EOS and LOS patients and average AAO. Cluster 2 and 4 were closer in terms of average AAO and enrichment of disease endotypes differentially modulated relative to NDC; the similarity of functional enrichment and differential TF activity suggest that cluster 4 may represent a more severe form of cluster 2. In contrast, cluster 1 demonstrated a later average AAO concomitant with the severity of co-expression modules or disease endotypes that were either less than the other two clusters (e.g., lower reactivation of REST or the M5–M11–M14 comodule) or in the opposite direction of other clusters (e.g., M4–M10–M18 comodule and synaptic function). The observation that some patient clusters within AD cohorts exhibit transcriptional dysregulation in the opposite direction of hallmark AD changes (e.g., loss of synaptic signaling and activation of inflammation) has been described in larger studies [9]. In our previous study of iPSC-derived neurons from patients harboring autosomal dominant AD-causing *PSEN1* mutations, we observed a stronger enrichment and simultaneous combination of common endotypes in mutations associated with earlier AAO (i.e., the *PSEN1*^*M146L*^ mutation) [10]. We surmise that the same trend may hold for EOS and LOS AD, although our results demonstrate that a larger cohort is needed to resolve the transcriptional delineation between these two types of the disease. It is possible that with a larger cohort, the AD profile type captured by cluster 1 (all but one patient with a diagnosed onset > age 60) would have a more substantial representation of LOS cases causing it to statistically separate from clusters with a strong representation of EOS cases (e.g., the cluster 4 AD profile type). Despite this cohort size limitation, our systems-level approach is able to deconvolute patient clusters and derive mechanistic insight for key disease endotypes identified previously in both autosomal-dominant and late-onset sporadic AD by us and others [9–11]. We anticipated that the primary visual cortex (Bm-17), a brain region thought to be less affected by AD pathology, may exhibit fewer transcriptomic changes than observed in regions canonically affected by AD; perhaps surprisingly, it displays analogous dysregulation of disease endotypes observed in other regions albeit with limited activation of inflammation, possibly due to a delayed onset of pathology [1, 12]. Our study characterized the transcriptome signature of whole Bm-17 tissue, which offers a broad insight into differential gene regulation due to sporadic AD, a region that likely contains a strong contribution from neuron dysregulation in the context of AD [13]. Looking forward, spatial transcriptomics as well as characterization of specific cell types (via single cell RNA-seq and ATAC-seq) will be needed to disentangle further the correlation between transcriptomic dysregulation and pathology and the complexity of gene regulatory control of disease endotypes arising from heterogenous EOAD and LOAD mechanisms, respectively.

## Methods

### Postmortem brain samples

Alzheimer’s disease (AD) and healthy, nondemented control (NDC) samples were obtained from the brain bank of samples preserved at the UC San Diego Shiley-Marcos Alzheimer’s Disease Research Center (UCSD ADRC), extracting tissue from the Brodmann Area 17 (Bm-17) of the occipital lobe (OL) per UC San Diego IRB approval. A total of 50 samples were selected: 8 NDC and 40 AD patient samples. The 40 AD samples were selected based on their lack of alternative diagnosis (e.g., Lewy Body Dementia, hippocampal sclerosis), APOE status (all AD and NDC samples were either *APOEε3/3* or the *APOEε3/4* genotype), and stratified into two groups based on the age at onset (AAO) of AD: early-onset, i.e., those with an AAO less than 60 years (n = 19), and late-onset, i.e. those with an AAO between 70 and 80 years (n = 21). Three cognitive evaluation scores, BIMC (Blessed Memory Information Concentration) [14], MMSE (Mini-Mental State Examination) [15], and Mattis’ DRS (Dementia Rating Scale) [16] were used to classify the selected patients as AD or NDC condition. All NDC patients had a BIMC score ≤ 4, MMSE score between 26 and 30, and an aggregate DRS score between 127 and 140. Each brain sample was also staged based on the concentration of Neurofibrillary Tangles (NFTs) in different brain regions, using a modified version of the staging scheme introduced by Braak and Braak. All AD samples were classified as BRAAK stage VI, while the NDC samples were classified at BRAAK stage I or II. Additional metadata for each sample was also collected for each AD sample: sex, AAO, age at death, and the concentration of neuritic plaques and tangles in the mid-frontal cortex (MF), inferior parietal cortex (IP), superior temporal cortex (ST), and hippocampus. As AD was ascertained to be the cause of death of all patients within this study, disease-specific survival (DSS) time was estimated by subtracting the age at diagnosis from age at death.

### RNA sequencing

RNA from brain samples was extracted using the RNeasy Lipid Tissue Mini kit (Qiagen Cat. 74804) according to the manufacturer’s protocol. Libraries were prepared for RNA-Seq using the TruSeq Stranded Total RNA Library prep kit (Illumina, Cat. RS-122-2303) by the Ribo-Zero ribosomal RNA reduction method (Illumina). Samples were sequenced at the UC San Diego Institute for Genomics Center sequencing core on an Illumina HiSeq4000 generating Paired-End, 75 bp reads with an average of 100 million reads per sample (Illumina, Cat. FC-410-1001).

### RNA-seq data processing and sample clustering

RNA-Seq data preprocessing was performed using the TrimGalore! package [17], removing sequencing adaptors and selecting for all paired-end reads above a quality score threshold (Phred Q > 20). Trimmed RNA-Seq reads were mapped to the GRCh38.p12 human transcriptome using kallisto v0.46.1 [18] with the options -bias and -rf-stranded. The R package *tximport* v1.8.0 [19] was used to summarize kallisto transcript abundancies to the gene level. A DGEList object was created from gene-level read counts using the *DGEList()* function from *edgeR* v3.30.3 [20]. Gene-level count filtering was applied using the *filterByExpr* function in *edgeR* for inclusion in further analysis, followed by count normalization using the TMM (Trimmed Mean of M-values) method using the function *calcNormFactors*. Hierarchical clustering was applied using the *factoextra* R package [21] to identify clusters of AD patients with similar transcriptional profiles. Genes were filtered by applying 10 counts per million (cpm) minimum threshold across all samples and expression corrected for sex using the *removebatcheffects* function in the *limma* R package. and Euclidean distance to compute pairwise similarity between the samples used to compare the dendrograms that ensure from either clustering analysis. The *voom* function from the *limma* v3.44.1 R package [22, 23] was used to model the mean–variance trend and capture gene-specific weights, which were subsequently used to fit a linear model to the count data including sex and RIN score. A contrast matrix was used to compare gene expression between the AD cluster subtypes and NDC samples, and empirical Bayesian statistics for the differential expression analysis was estimated using the *eBayes* function from *limma*. Genes with an FDR-adjusted p-value of less than 0.05 were deemed as being differentially expressed between each AD condition and NDC. To determine whether quality surrogate variable analysis (qSVA) would be useful to apply to the AD cluster subgroups to correct for degradation associated with RIN score, DEqual plots, a diagnostic plot that shows the correlation in differential expression t-statistics between AD-induced and degradation-induced differential expression, were generated to quantify degradation in different sample clusters [3].

### Geneset enrichment and transcription factor activity analysis

Geneset enrichment analysis was performed by two weighted approaches: competitive, directional enrichment using the *fgseamultilevel* function in the *fgsea* [24] R package and non-competitive, unidirectional enrichment using the *tmodCERNOtest* function in the *tmod* [25] R package, both with The Gene Ontology-Biological Process (GOBP) [26], Reactome [27], and Hallmark [28] ontology geneset databases as well as the StringDB [29] protein–protein interaction (PPI) database. For *fgsea*, genes were ranked by the *limma* t-statistic, while for CERNO genes were ranked by minimum significant distance (msd). Transcript reads for ISMARA motif activity analysis were filtered using the *filterByExpr* function in *edgeR.* To determine a directional *z*-score for each enriched motif identified, the differential *z*-score for each given motif between each AD cluster and NDC was multiplied by the sign of the Pearson correlation between each motif and its target genes. In cases where ISMARA did not calculate a Pearson correlation, literature evidence of the activator or repressor function of the given TF was used.

### Co-expression module analysis

Modules of co-expressed genes across NDC and AD samples (clusters 1, 2, and 4) were identified using the *CEMiTool* R package [7] on genes with > 10 cpm expression across all samples. Prior to module detection, counts were transformed using the *voomwithqualityweights* function in the *limma* R package with the parameters directed = TRUE and cor_method = pearson. Gene Set Variation Analysis (GSVA) [8] using the *GSVA* R package was subsequently performed on the resulting 22 co-expression modules across NDC and AD samples. To determine the enrichment of CEMiTool modules in each AD cluster relative to NDC, GSVA scores were used as an input for the *camera* [30] enrichment function in the *limma* R package*.* Hypergeometric enrichment of CEMiTool modules or comodules was performed using the *tmodhgtest* function in the *tmod* R package [25] and Gene Ontology: BP [26], Hallmark [28], or Reactome [27] ontology gene set databases as well as ENCODE-ChEA Consensus [31, 32] and ReMap [33] TF-gene target databases. The StringDB PPI database v10 [29] was filtered for high-confidence interactions sourced from a) databases and b) literature physical interactions and subset for the genes in a given module.

## Supplementary Information

**Additional file 1.** Supplementary figures S1 and S2.

**Additional file 2.** Brain metadata, RNA-seq counts, and hypergeometric enrichment using the GO:BP geneset library of the top 20 modules identified by CEMiTool.

## Data Availability

RNA-seq data is available at the NCBI GEO under the accession GSE203206. RNA-seq counts and pathology metadata can be found in Additional files 1 and 2.

## References

[1] Haroutunian V, Katsel P, Schmeidler J (2009). Transcriptional vulnerability of brain regions in Alzheimer’s disease and dementia. Neurobiol Aging.

[2] Cacace R, Sleegers K, Van Broeckhoven C (2016). Molecular genetics of early-onset Alzheimer’s disease revisited. Alzheimers Demen.

[3] Jaffe AE, Tao R, Norris AL, Kealhofer M, Nellore A, Shin JH (2017). qSVA framework for RNA quality correction in differential expression analysis. PNAS Nat Acad Sci.

[4] Nygaard HB (2018). Targeting Fyn kinase in Alzheimer’s disease. Biol Psychiatr Elsevier.

[5] Zhang M, Dilliott AA, Khallaf R, Robinson JF, Hegele RA, Comishen M (2019). Genetic and epigenetic study of an Alzheimer’s disease family with monozygotic triplets. Brain.

[6] Shigemizu D, Mori T, Akiyama S, Higaki S, Watanabe H, Sakurai T (2020). Identification of potential blood biomarkers for early diagnosis of Alzheimer’s disease through RNA sequencing analysis. Alzheimers Res Ther.

[7] Russo PST, Ferreira GR, Cardozo LE, Bürger MC, Arias-Carrasco R, Maruyama SR (2018). CEMiTool: a bioconductor package for performing comprehensive modular co-expression analyses. BMC Bioinformat.

[8] Hänzelmann S, Castelo R, Guinney J (2013). GSVA: gene set variation analysis for microarray and RNA-Seq data. BMC Bioinformat.

[9] Neff RA, Wang M, Vatansever S, Guo L, Ming C, Wang Q (2021). Molecular subtyping of Alzheimer’s disease using RNA sequencing data reveals novel mechanisms and targets. Sci Adv Am Assoc Adv Sci.

[10] Caldwell AB, Liu Q, Schroth GP, Galasko DR, Yuan SH, Wagner SL (2020). Dedifferentiation and neuronal repression define familial Alzheimer’s disease. Sci Adv Am Assoc Adv Sci.

[11] Mertens J, Herdy JR, Traxler L, Schafer ST, Schlachetzki JCM, Böhnke L, et al. Age-dependent instability of mature neuronal fate in induced neurons from Alzheimer’s patients. Cell Stem Cell. 2021;28:1533–48.e6. 10.1016/j.stem.2021.04.004 PMC8423435 33910058

[12] Haroutunian V, Perl DP, Purohit DP, Marin D, Khan K, Lantz M (1998). Regional distribution of neuritic plaques in the nondemented elderly and subjects with very mild Alzheimer disease. Arch Neurol.

[13] Wang M, Roussos P, McKenzie A, Zhou X, Kajiwara Y, Brennand KJ (2016). Integrative network analysis of nineteen brain regions identifies molecular signatures and networks underlying selective regional vulnerability to Alzheimer’s disease. Genome Med.

[14] Blessed G, Tomlinson BE, Roth M (1968). The association between quantitative measures of dementia and of senile change in the cerebral grey matter of elderly subjects. Br J Psychiatry.

[15] Folstein MF, Folstein SE, McHugh PR (1975). “Mini-mental state”. A practical method for grading the cognitive state of patients for the clinician. J Psychiatr Res..

[16] Mattis S. Mental Status examination for organic mental syndrome in the elderly patient. In: Bellack L, Karusu TB, editors. Geriatric psychiatry. Grune & Stratton, New York; 1976. p. 77–121.

[17] Martin M (2011). Cutadapt removes adapter sequences from high-throughput sequencing reads. EMBnet J..

[18] Bray NL, Pimentel H, Melsted P, Pachter L (2016). Near-optimal probabilistic RNA-seq quantification. Nat Biotechnol.

[19] Soneson C, Love MI, Robinson MD (2016). Differential analyses for RNA-seq: transcript-level estimates improve gene-level inferences. F1000Research..

[20] Robinson MD, McCarthy DJ, Smyth GK (2010). edgeR: a Bioconductor package for differential expression analysis of digital gene expression data. Bioinformatics.

[21] Kassambara A, Mundt F. Factoextra: extract and visualize the results of multivariate data analyses. R package [Internet]. Available from: https://CRAN.R-project.org/package=factoextra.

[22] Law CW, Chen Y, Shi W, Smyth GK (2014). Voom: precision weights unlock linear model analysis tools for RNA-seq read counts. Genome Biol.

[23] Ritchie ME, Phipson B, Wu D, Hu Y, Law CW, Shi W (2015). limma powers differential expression analyses for RNA-sequencing and microarray studies. Nucleic Acids Res.

[24] Korotkevich G, Sukhov V, Sergushichev A. Fast gene set enrichment analysis. bioRxiv. Cold Spring Harbor Laboratory; 2019;060012.

[25] Weiner 3rd J, Domaszewska T. tmod: an R package for general and multivariate enrichment analysis [Internet]. PeerJ Inc.; 2016 Sep. Report No.: e2420v1. Available from: https://peerj.com/preprints/2420.

[26] Ashburner M, Ball CA, Blake JA, Botstein D, Butler H, Cherry JM (2000). Gene Ontology: tool for the unification of biology. Nat Genet.

[27] Croft D, O’Kelly G, Wu G, Haw R, Gillespie M, Matthews L (2011). Reactome: a database of reactions, pathways and biological processes. Nucleic Acids Res.

[28] Liberzon A, Birger C, Thorvaldsdóttir H, Ghandi M, Mesirov JP, Tamayo P (2015). The Molecular Signatures Database (MSigDB) hallmark gene set collection. Cell Syst.

[29] Szklarczyk D, Franceschini A, Wyder S, Forslund K, Heller D, Huerta-Cepas J (2015). STRING v10: protein–protein interaction networks, integrated over the tree of life. Nucleic Acids Res.

[30] Wu D, Smyth GK (2012). Camera: a competitive gene set test accounting for inter-gene correlation. Nucleic Acids Res.

[31] Chen EY, Tan CM, Kou Y, Duan Q, Wang Z, Meirelles GV (2013). Enrichr: interactive and collaborative HTML5 gene list enrichment analysis tool. BMC Bioinformatics.

[32] Kuleshov MV, Jones MR, Rouillard AD, Fernandez NF, Duan Q, Wang Z (2016). Enrichr: a comprehensive gene set enrichment analysis web server 2016 update. Nucleic Acids Res.

[33] Chèneby J, Gheorghe M, Artufel M, Mathelier A, Ballester B (2018). ReMap 2018: an updated atlas of regulatory regions from an integrative analysis of DNA-binding ChIP-seq experiments. Nucleic Acids Res.

[34] Reitz C, Rogaeva E, Beecham GW (2020). Late-onset vs nonmendelian early-onset Alzheimer disease: a distinction without a difference?. Neurol Genet.

[35] Wingo TS, Lah JJ, Levey AI, Cutler DJ (2012). Autosomal recessive causes likely in early-onset Alzheimer disease. Arch Neurol.

[36] Fulton-Howard B, Goate AM, Adelson RP, Koppel J, Gordon ML, Barzilai N (2021). Greater effect of polygenic risk score for Alzheimer’s disease among younger cases who are apolipoprotein E-ε4 carriers. Neurobiol Aging.

[37] Caldwell AB, Liu Q, Zhang C, Schroth GP, Galasko DR, Rynearson KD, et al. Endotype reversal as a novel strategy for screening drugs targeting familial Alzheimer’s disease. Alzheimer's Demen. 2022;1–14. 10.1002/alz.12553. 10.1002/alz.12553 PMC9787711 35084109

