# Supplementary material for: Transcriptomic profiling of sporadic Alzheimer’s disease patients
Source: Mol Brain. 2022 Oct 12;15:83. doi: 10.1186/s13041-022-00963-2 (PMC9559068; doi:10.1186/s13041-022-00963-2)
Supplement: Supplementary file 1 — Additional file 1. Supplementary figures S1 and S2. [file 13041_2022_963_MOESM1_ESM.pdf]

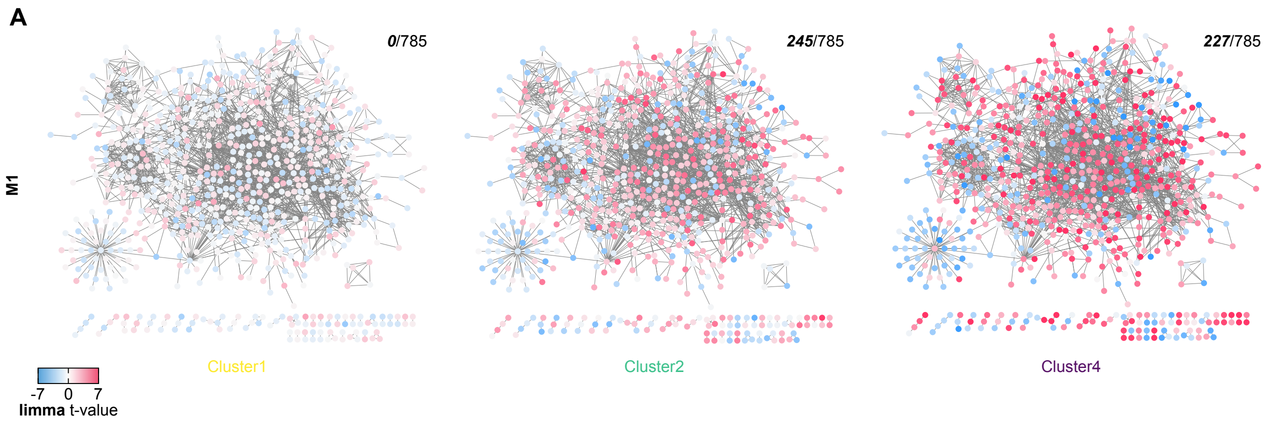

B

Eenriched

Pathways

Locomotion
adj. *P*: 5.87x10^{-13}
Blood vessel morphogenesis
adj. *P*: 1.23x10^{-11}
Neurogenesis
adj. *P*: 7.14x10^{-11}

TFs

CEBPB
adj. *P*: 2.41x10^{-14}
RUNX2
adj. *P*: 2.68x10^{-13}
TEAD4
adj. *P*: 4.07x10^{-11}

Figure S1

A. StringDB PPI interaction networks for module M1 across the three clusters; genes color-coded by limma t-value; upper right subpanel indicates number of DEGs within the module for each AD cluster. **B.** Highly enriched pathways and TFs for each comodule (hypergeometric test).

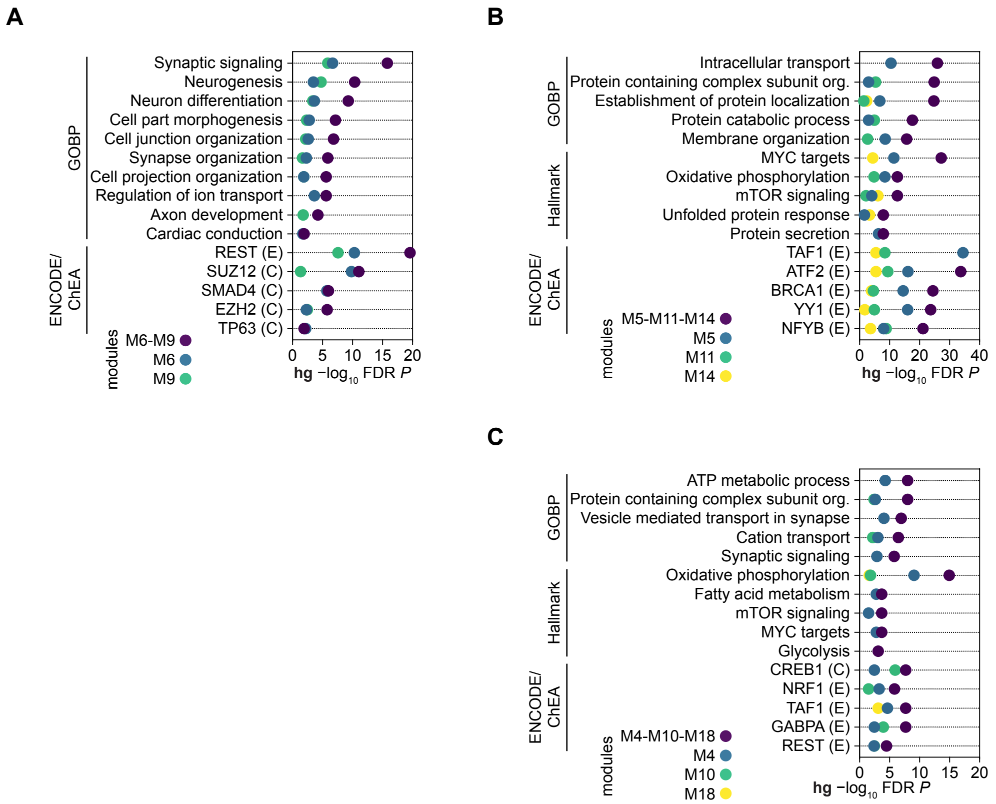

Figure S2

A-C Hypergeometric enrichment (hg) of modules and combined comodules using the Gene Ontology – Biological Process (GOBP), Hallmark, and ENCODE-ChEA Consensus databases for comodule **A** M6-M9, **B** M5-M11-M14, **C** M4-M10-M18.
